# Angora Wool Asthma in Textile Industry

**DOI:** 10.1155/2012/358271

**Published:** 2012-09-23

**Authors:** Pietro Sartorelli, Riccardo Romeo, Giuseppina Coppola, Roberta Nuti, Valentina Paolucci

**Affiliations:** Unit of Occupational Medicine and Toxicology, University of Siena, 16 Bracci Avenue, 53100 Siena, Italy

## Abstract

Up to now the exposures to hair and skin derivatives of animals have not yet been the subject of systematic studies. The observation of a clinical case has provided the opportunity for a review of the literature. The inpatient was a 49-year-old man, a carder in a textile factory, exposed to angora wool. He noticed the appearance of dyspnea during working hours. There was no eosinophilia in blood, and the results of pulmonary function tests were normal. The nonspecific bronchial provocation test with methacholine demonstrated an abnormal bronchial reactivity. The challenge test with angora wool was positive (decrease in FEV1 of more than 40%) as well as total IGE and specific IgE to rabbit epithelium (433 KU/l and 12.1 KUA/l, resp.). Several sources of allergens were found in the rabbit, and the main allergen was represented by proteins from epithelia, urine, and saliva. Most of these proteins belong to the family of lipocalin, they function as carriers for small hydrophobic molecules (vitamins and pheromones). If the diagnosis of occupational asthma caused by animal hair and skin derivatives may be relatively easy by means of the challenge test, defining etiology is complicated because of the lack of in vitro tests.

## 1. Introduction

Defining the pathogenesis, prevention, and management of occupational asthma is an involved process. Diagnosis of occupational asthma requires the integration of a multiplicity of data such as respiratory function test, nonspecific bronchial hyperreactivity test, occupational challenge test (OCT), and the timing of symptoms in relation to the occupational activities. Cutaneous tests are particularly helpful in IgE-mediated asthma in relation to the inhalation of protein aeroallergens. For haptens, because they require prior coupling to a protein carrier, they cause problems in laboratory tests. The OCT represents the golden standard for etiological diagnosis of occupational asthma. The substances responsible for occupational asthma are mainly animal allergens, vegetable agents, and chemicals. In addition to major inducers of occupational asthma, there are other agents whose importance is still difficult to understand. Among these there are high molecular weight substances such as hair and skin derivatives of animals. The exposed professional categories are mainly farmers and workers in charge of laboratory animals [1]. Up to now these exposures have not yet been the subject to systematic studies as literature only reports cases of laboratory animal allergy and domestic exposures.

There are various types of angora rabbit. Each breed produces different fur. Angora wool harvesting occurs up to three times a year and is collected by shearing or from the molting fur. This type of wool is commonly used in the textile industry for apparel such as sweaters and suits. The observation of a case of angora wool asthma has provided the opportunity for a review of the literature.

## 2. Case Report

The in-patient was a 49-year-old man, a carder in a textile factory, mainly exposed to angora wool. He noticed the appearance of dyspnea during working hours. There was no eosinophilia in his blood and the results of pulmonary function tests were normal compared to CECA 1971 reference values. The nonspecific bronchial provocation test with methacholine through dosimeter [2] demonstrated an abnormal bronchial reactivity. A prick test of 12 common allergens (grass, composite, pellitory, olives, cypress, alternaria, dermatophagoides farinaceous and pteronyssinus, aspergillus fumigatus, dog, cat and horse, as well as the negative and positive controls; Lofarma Laboratories, Milan, Italy) were positive to pollens. The results of the prick tests were considered positive when they provoked a rash with an average diameter of ≥5 mm [3].

**Figure 1:**
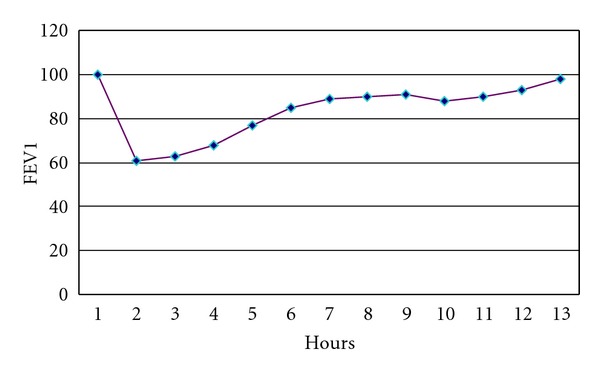
Occupational challenge test with angora wool.

Measurement of total and specific IgE (CAP System, Phadia, Uppsala, Sweden) showed a significant positive finding of total IGE (433 KU/l) and a positive IgE to rabbit epithelium (12.1 KUA/l). After the test with a control substance (talc powder) OCT was carried out by tipping angora wool into a 8 m^3^ ventilated room and assessing FEV1 for 8 hours (Figure 1). Exposure to angora wool was stopped after 15 minutes due to the onset of coughing and dyspnea with a decrease in FEV1 of more than 40%. The nasal lavage cell counts after OCT showed an increased percentage of neutrophils (96% neutrophils, 4% epithelial cells).

The patient was also suffering from hands dermatitis. Allergens from the standard tray (SIDAPA) and the textile industry tray were used for patch testing (Firma, Florence, Italy) by the Italian public employers' liability insurance (INAIL) showing a skin positivity to balsam of Peru (++), dimethylaminopropylamine (++), benzalkonium chlorure (++), and triethanolamine (+). An occupational allergic contact dermatitis was diagnosed because triethanolamine is used as surface-active agent in textile industry.

## 3. Literature Analysis

Recently two PubMed search strings determinants (one more specific, the other more sensitive) have been used to retrieve information on the possible association between occupational risk factors and some pathologies [4]. Using *wool asthma, asthma and rabbit, asthma and rabbit hair, rabbit allergens and lipocalin*, 116 papers were found with the specific string (25 pertinent and 1 highly pertinent) and 197 with the sensitive one (3 pertinent and 5 highly pertinent not retrieved by the specific string). Articles mainly regarding workers in charge of laboratory animals were found. Cases of asthma due to hair and skin derivatives of animals which occurred in the textile industry were not reported in literature. In all 1 case of angora wool asthma was only found [5].

Several allergenic proteins from rabbit have been recognized by crossed immunoelectrophoresis but have not been characterized. Understanding of these important occupational allergens will allow the development of a new diagnostic approach for affected workers and others who may be at risk. Baker et al. [6] recognized as allergens 26 protein bands in the three extracts: 12 in saliva, 7 in urine, and 7 in fur. This was the first evidence that allergens from the rabbit are members of the lipocalin superfamily of proteins, suggesting that similar mechanisms may be involved in eliciting the allergic response to rabbits. The 18 kDa allergen from saliva may be the previously named rabbit allergen, Ory c 1. The major laboratory animal allergens are carried on small particles that are both capable of remaining airborne for extended periods and penetrating the lower airways of exposed workers [7]. Lipocalins share common biological functions, predominantly related to the transport of small hydrophobic molecules, such as vitamins and pheromones. Immune reactivity to lipocalin allergens is not well known. Three of their epitopes were colocalized in their structurally conserved regions. Interestingly, one of the epitopes was recognized by the T cells of all patients and the computer predictions suggested that there would be an epitope in the corresponding parts of human endogenous lipocalins [6, 8]. Experimental studies on sensitizing properties of these molecules were only carried out on laboratory animal allergies [9, 10]. Lipocalins share sequence homology with antigens of the parasitic agent that causes schistosomiasis. The fact that parasite infections also trigger IgE antibody responses may account for the development of laboratory animal allergies in subjects who have never had any previous allergy [9].

## 4. Discussion

Analysis of the case gives the impression that the detection of specific antigens responsible of asthma in workers exposed to dermal derivatives and skin of animals is quite complex. Even if in the specific case the diagnosis was relatively simple by using OCT, etiology is not easily understandable because of the lack of immunological tests referring asthma to specific occupational compounds. At the moment OCT represents the golden standard for diagnosis and only prick tests can be used in prevention to single out subjects susceptible to developing an allergic respiratory disease to hair and skin derivatives of animals. In textile industry individuals with IgE response to rabbit epithelium should be considered susceptible to developing angora wool asthma.
